# Rab14 Act as Oncogene and Induce Proliferation of Gastric Cancer Cells via AKT Signaling Pathway

**DOI:** 10.1371/journal.pone.0170620

**Published:** 2017-01-20

**Authors:** Bo Guo, Wenjing Wang, Zhenghao Zhao, Qian Li, Kaiyue Zhou, Lingyu Zhao, Lumin Wang, Juan Yang, Chen Huang

**Affiliations:** 1 Department of cell Biology and Genetics, School of Basic Medical Sciences, Xi'an Jiaotong University Health Science Center, Xi'an, Shaanxi, P. R. China; 2 Department of Hepatobiliary Surgery, First Affiliated Hospital, Xi'an Jiaotong University, Xi'an, Shaanxi, P. R. China; 3 Program in Plant Biology and Conservation, Biological Sciences, Weinberg College of Arts and Sciences, Northwestern University, Evanston, Illinois, United States of America; 4 Key Laboratory of Shaanxi Province for Craniofacial Precision Medicine Research, College of Stomatology, Xi'an Jiaotong University, Xi’an, Shaanxi, P. R. China; 5 Key Laboratory of Environment and Genes Related to Diseases (Xi’an Jiaotong University), Ministry of Education of China, Xi’an, Shaanxi, P. R. China; University of Navarra, SPAIN

## Abstract

Rab14 is a member of RAS oncogene family, and its dysfunction has been reported to be involved in various types of human cancer. However, its expression and function were still unclear in gastric cancer. The aim of this study was to investigate the function and mechanism of Rab14 in gastric cancer cell lines. Quantitative real-time PCR (qRT-PCR) was performed in 17 gastric adenocarcinoma tissues and 4 cell lines to detect the expression of Rab14. 3-(4, 5-dimethylthiazol-2-yl)-2, 5-diphenyl-tetrazolium bromide (MTT), colony formation and flow cytometry assays were employed to determine the proliferative ability, cell cycle transition and apoptosis *in vitro* in BGC-823 or SGC-7901 cells. Western blot was performed to investigate the pathways and mechanism of Rab14 regulation. In this study, we show that Rab14 presents a significant up-regulated expression among the paired tissue samples and cell lines in gastric cancer. When we overexpressed Rab14 in SGC-7901 cells or silenced Rab14 in BGC-823 cells, we found that Rab14 could modify cell growth, cell cycle or apoptosis, which accompanied with an obvious regulation of CCND1, CDK2 and BAX involving in AKT signaling pathway. In conclusion, this study provides a new evidence on that Rab14 functions as a novel tumor oncogene and could be a potential therapeutic target in gastric cancer.

## Introduction

Gastric cancer (GC) is the most common cause of cancer-related death worldwide [1]. China is one of the countries with a highest GC incidence rates accounting for over 40% of all GC cases worldwide [2, 3]. The tumorigenesis of GC is a multistep process, which results from activation of oncogenes or inactivation of tumor suppressor genes [4]. Numerous studies have indicated that the development and progression of GC are due to miss-regulation of many related genes such as p53 [5], AKT [6] and PTEN [7]. Therefore, a better understanding of the molecular mechanisms involved in GC formation and development will be beneficial to discover novel therapeutic targets and develop effective strategies for the treatment of GC.

In the past few decades, Rab-GTPase-directed pathways have begun to emerge as key events in tumor proliferation [8]. RAB proteins have been reported to play a vital role in vesicle trafficking [9], signal transduction [10] and receptor recycling [11]. For example, Kawauchi et.al showed that Rab5-dependent endocytic transport of cadherin proteins acts as receptor trafficking for controlling cell-cell adhesions, which is important during vertebrate gastrulation and brain development [12]. Rab14, as a member of RAS oncogene family, is the last member of the Rab11 subfamily and identified together with Rab5, Rab7 and Rab1 in the proteome of endosomes isolated from migrating cells [13]. To date, there has only been few reports on the association between Rab14 and human cancers. Zhang et.al have identified Rab14 protein as a possible tumor marker for lung cancer [14]. Besides, Wang et.al. found that protein expression of Rab14 showed strongly positive compare to corresponding non-tumor lung tissues in NSCLC. In addition, inhibition of Rab14 with RNA interference could significantly suppress cell proliferation, which also indicated that Rab14 function as an oncogene in human NSCLC [15]. Nevertheless, the role of Rab14 in the pathogenesis of gastric cancer is still not clear. Thus, the identification and characterization of Rab14 is critical to understand its function in GC progression and development.

In this study, we analyzed the expression profiles of Rab14 in GC tissue and cell lines. We also investigated the role and the underlying molecular mechanisms of Rab14 in GC. The goal of this study is to clarify the expression and functions of Rab14 in GC and offer a potential targets for diagnosis and therapy for GC.

## Materials and Methods

### Human tissue samples and cell lines

Human tissue samples of gastric cancer and the matched non-tumor gastric tissues (at least 5cm away from the tumor edge) were obtained from patients who had undergone surgical gastric resection at the First Affiliated Hospital of Xi'an Jiaotong University (Informed consent was obtained from each patient and was approved by the Institute Research Ethics Committee at Cancer Center, Xi'an Jiaotong University). The human gastric cancer cell lines (BGC-823, AGS, MKN-45 and SGC-7901) and immortalized human gastric epithelial mucosa cell line (GES-1) were maintained in the Key Laboratory of Environment and Genes Related to Diseases at Xi'an Jiaotong University College of Medicine. Cells were cultured in Dulbecco's Modified Eagle Medium (PAA, Australia), supplemented with 10% Fetal Bovine Serum (PAA, Australia) and 1% Penicillin/Streptomycin in humidified atmosphere with 5% CO_2_/ 95% air at 37°C.

### RNA extraction, cDNA synthesis, and quantitative real-time PCR

Total RNA was isolated from prepared GC samples or cells with TRIzol reagent (Life Technologies, USA), and cDNA was then synthesized with PrimeScript RT Reagent Kit according to the manufacturer's protocol (TAKARA, Japan). Quantitative real-time PCR (qRT-PCR) was performed using SYBR Green Ex Taq^™^ II (TAKARA, Japan), and PCR-specific amplification reactions were conducted in the IQ5 Optical System real-time PCR machine (BIO-RAD, USA). The relative expression of genes was calculated with the 2^-(DDCt)^ method [16]. The primers used are listed below (qRT-PCR, Rab14-F 5’-GCAGATTTGGGATACAGCAGGG-3’, Rab14-R 5’- CAGTGTTTGGATTGGTGAGATTCC-3’; GAPDH-F 5’-TGAAGGTCGGAGTCAACGGATT-3’, GAPDH-R 5’-CCTGGAAGATGGTGATGGGATT-3’).

### Plasmid construction and cell transfection

To overexpress Rab14 in SGC-7901 cells, the cDNAs encoding human Rab14 were amplified (Rab14 Forward: 5’-TCCGCTCGAGATGGCAACTGCACCATAC-3’; Rab14 Reverse: 5’-ATGGGGTACCGAGCAGCCACAGCCTTCTCTC-3’) and cloned into the GV-141 expression vector MCS with Not I and Kpn I restriction sites (GV-Rab14). Empty vector GV-141 was used as the control in the Gain-of-Function experiments (GV-CON); To silence Rab14 in BGC-823 cell, a short hairpin RNA was designed based on the sequence: 5’-TGCAAGGAATCTCACCAAT-3’, and was cloned into GV-112 expression vector (sh-Rab14). Empty vector GV-112 was used as the control in the Loss-of-Function experiments (sh-CON). The plasmids were constructed and verified by Shanghai Genechem Co., LTD.

The plasmid DNAs were transfected with X-treme GENE HP DNA Transfection Reagent (Roche, USA) according to the manufacturer's instructions, and the transfection efficiency was determined by qRT-PCR.

### Cell proliferation assay

3-(4, 5-dimethylthiazol-2-yl)-2, 5-diphenyl-tetrazolium bromide (MTT) assay was performed to investigate the effect of Rab14 overexpressing in SGC-7901 cells or Rab14 silencing in BGC-823 cells on the proliferation ability. Cells were seeded at a density of 1×10^4^ cells/well into 96-well plates and transfected the following day. 10μl of MTT solutions (5mg/ml) were added to wells (Sigma, USA) at 24, 48 and 72 h, respectively, and continued incubating for 2~4 h at 37°C in 5% CO_2_. After that, the supernatant was discarded while 150μl of Dimethyl Sulfoxide (DMSO) was added to the cells. Sample absorbance was measured at A450 nm by a high-throughput universal microplate assay (BMG Lab Technologies, Germany).

### Cell cycle assay

To determine the distribution of cells in the cell cycle, SGC-7901 or BGC-823 cells were cultured in 12-well plates at a density of 1×10^6^ cells per well in triplicate. 48 hours after transfection with plasmid, cells were harvested by trypsinization, washed with PBS and fixed in 75% ice-cold ethanol overnight at 4°C. After washed in PBS and incubated with 300μl of staining solution (20 mg/ml Propidium Iodide and 10 U/ml RNase A) for 30 minutes at room temperature. Cell-cycle distributions were assessed by fluorescence-activated cell sorting based on flow cytometer (Becton-Dickinson, USA).

### Cell apoptosis assay

To explore the proportion of the apoptotic cells, cell apoptosis analysis was performed with Annexin-V FITC Apoptosis Detection Kit (Life Technologies, USA) according to the manufacturer's instructions. SGC-7901 or BGC-823 cells were seeded into 12-well plates at a density of 1×10^6^ cells per well in triplicate, and transfected for 48 hours, and then examined by a flow cytometer. The apoptosis populations were determined by ModFit software.

### Colony formation assay

Stably transfected cells were seeded at a density of 5×10^3^ per 6-well plate, incubated for 1~2 weeks. The cell colony was stained with 0.5% crystal violet for 30 minutes. The stained cell clones were taken pictures after excess dye was rinsed off twice with PBS. Then 33% acetic acid was added into wells and shake for 30 minutes. The microplates were read at A450nm to obtain OD values.

### Western blot analysis

Western blot was employed to determine the regulated gene by Rab14 in the AKT pathway. Total proteins were extracted with RIPA lysis buffer (Wolsen, China) from cells harvested 48 hours after transfection, and were separated by 10% SDS polyacrylamide gels. They were then electrophoretically transferred to polyvinylidene difluoride membrane (Millipore, USA). After the samples were incubated with primary antibodies at 4°C overnight and secondary antibodies for 2h at room temperature, then were visualized with enhanced chemiluminescence detection system (UVP, USA). The primary antibodies for the differentially expressed gene used were as following dilutions: rabbit polyclonal anti–Rab14 (working dilutions: 1:200, #15662-1-AP, Proteintech, China), rabbit mAb anti-Akt (working dilutions: 1:1,000, #9272, Cell Signal Technology, USA), rabbit mAb anti–phospho-AktSer473 (; working dilutions: 1:2,000, #4060, Cell Signal Technology, USA), rabbit mAb anti-CCND1 / p-CCND1 (working dilutions: 1:1,000, #2978 / #3300, Cell Signal Technology, USA), rabbit mAb anti-CDK2 (working dilutions: 1:500, #2546, Cell Signal Technology, USA), rabbit mAb anti-Bax (working dilutions: 1:1,000, #5023, Cell Signal Technology, USA) and mouse monoclonal anti-GAPDH (working dilutions: 1:2,000, #8795, Sigma-Aldrich, USA).

### Statistical analysis

Each experiment was repeated at least three times independently. Data were presented as mean±SD and analyzed using the PASW Statistics 18 software (SPSS, USA). Differences between 2 groups were calculated with the Student-t or χ2 test. P<0.05 was considered to be statistically significant.

## Results

### Rab14 is up regulated in GC tissue and cell lines

**Fig 1:**
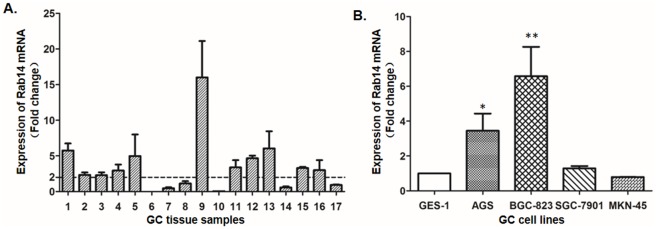
Rab14 is up-regulated in gastric cancer tissues and cell lines. (A) qRT-PCR was performed to examine Rab14 expression in 17 paired human gastric cancer and adjacent normal tissues. (B) qRT–PCR analysis of Rab14 expression in normal GES-1 cells and four gastric cancer cell lines. The expression of Rab14 was normalized to GAPDH. Data were analyzed using a 2^-ΔΔCt^ approach. One-way analysis of variance was adopted to compare Rab14 expression and all data are shown as mean±s.d. for three independent experiments (*P<0.05).

To determine whether Rab14 was a putative oncogene in GC, qRT–PCR analysis was performed to detect the expression levels of Rab14 in GC tumor and matched non-tumor tissues. As shown in Fig 1A, among the 17 paired samples, 11 of them (65%) exhibited a higher (Fold change≥2) expression of Rab14 in gastric cancer tissues compared to the matched non-tumor gastric tissues. The average fold change of Rab14 was 3.288 (±3.830). Furthermore, we measured the expression levels of Rab14 in four human GC cell lines (AGS, BGC-823, MKN-45 and SGC-7901) and a normal gastric epithelial cell line (GES-1). Compared to GES-1, the expression level of Rab14 was up-regulated in SGC-7901 cells (fold change = 1.29) particularly significantly higher in AGS (fold change = 3.45) and BGC-823 (fold change = 6.58) cells lines, except MKN-45 (fold change = 0.79) (Fig 1B). Considering the transfection efficiency for following study, we used SGC-7901 cells to overexpress Rab14 and BGC-823 cells to silence Rab14 with the aim of investigating its role in the development and progression of GC.

### Rab14 can induce GC cell proliferation

**Fig 2:**
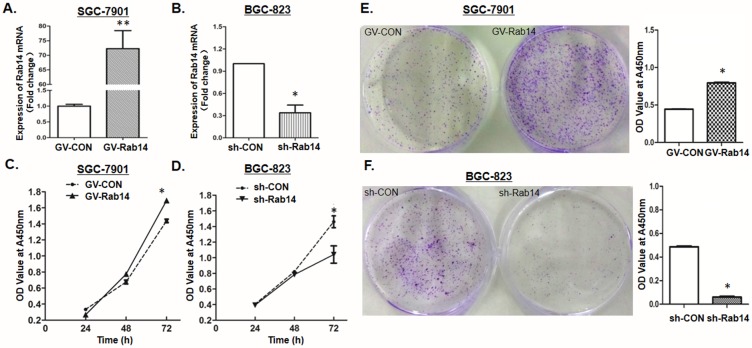
Rab14 could remarkably induce cell proliferation of GC cells. (A/B) Expression levels of Rab14 was measured by qRT-PCR to confirm the transfection efficiency of overexpressing Rab14 in SGC-7901 cells or silencing Rab14 in BGC-823 cells. (C/D) The effects of Rab14 on cell proliferation were determined by MTT assay at 24, 48 and 72 h in SGC-7901 cells or BGC-823 cells after transfection with GV-Rab14 or sh-Rab14 and their control vectors, respectively. (E/F) Representative results of colony formation in SGC-7901 or BGC-823 cells, respectively (*P<0.05). The absorbance of solution (OD values) was read at 450 nm using a microtiter plate reader.

To validate that Rab14 may function as a tumor oncogene, the effects of Rab14 up- or down-regulation on the proliferation of GC cells were studied in vitro. First, expression levels of Rab14 were determined by qRT-PCR in both SGC-7901 cells transfected with GV-Rab14 and BGC-823 cells transfected with sh-Rab14 (Fig 2A and 2B), indicating that Rab14 were successfully overexpressed in SGC-7901 cells and silenced in BGC-823 cells through plasmid transfected. Thereafter, MTT and colony formation assays were performed to detect cell viability. As shown in Fig 2C and 2D, overexpressing Rab14 in SGC-7901 cells can greatly induce cell viability, while BGC-823 cells transfected with sh-Rab14 vector were observed to grow more slowly than those cells transfected with sh-CON. Colony formation assay was performed as well. As shown in the results, GV-Rab14 could promote cell colony formation in SGC-7901 cells (Fig 2E). In contrast, Rab14 silencing resulted in remarkably fewer and smaller colony numbers in BGC-823 cells (Fig 2F). Thus, the results of colony formation assay were consistent with those of MTT assay and further indicated that Rab14 could remarkably induce GC cell proliferation.

### Rab14 contributes to G0/G1-S phase transition in GC cell lines

**Fig 3:**
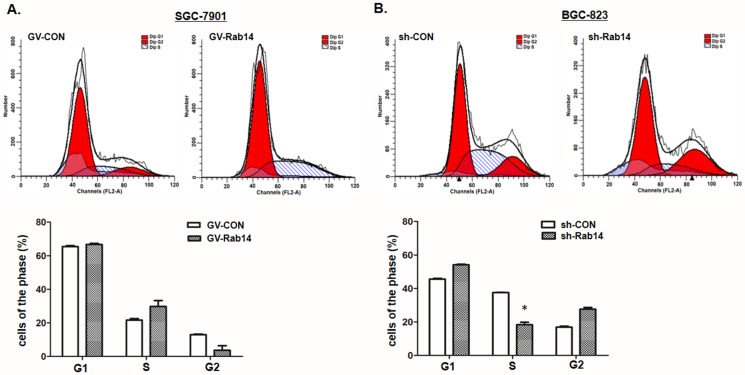
Rab14 contributes to G0/G1-S phase transition in GC cells. Cell cycle detected by Propidium-iodide staining with flow cytometry in SGC-7901 or BGC-823 cells 48 h after transfection with GV-Rab14 or sh-Rab14, respectively. Histogram represented the percentage of cells in G0–G1, S and G2–M cell-cycle phases, (*P<0.05).

To further investigate the mechanisms by which Rab14 promotes cell proliferation, flow cytometry analysis was chosen to analyze the cell cycle of GC cell lines after transfection with GV-Rab14 or sh-Rab14. As shown in Fig 3A, GV-Rab14 transfected into SGC-7901 cells led to a slight cycle arrest in S phases compared to GV-CON transfected group. In addition, silence of Rab14 resulted in a remarkable accumulation of G0/G1-population in the BGC-823 cells (Fig 3B), suggesting that Rab14 can leads a G0/G1–S phase transition in GC cells.

### Silencing of Rab14 induce apoptosis in BGC-823 cell lines

**Fig 4:**
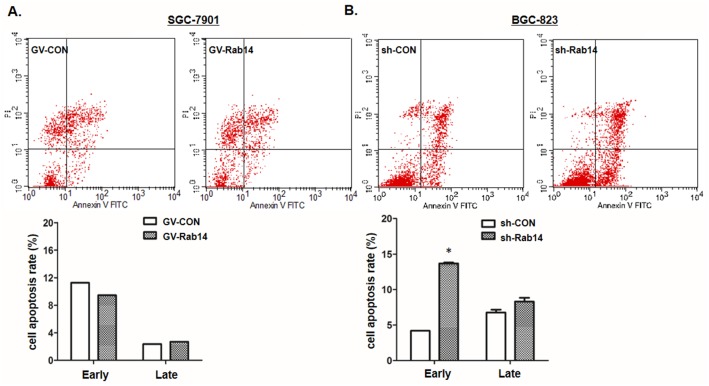
Silence of Rab14 induce apoptosis in GC cells. Cell apoptosis were detected by Annexin-V/Propidium Iodide combined labeling with flow cytometry in SGC-7901 or BGC-823 cells 48 h after transfection with GV-Rab14 or sh-Rab14, respectively. Dot plot showed of SGC-7901 cells (A) and BGC-823 cells (B). The total percentage of cell death in sh-Rab14 treated BGC-823 cells, including early apoptotic cells stained as Annexin V-positive(lower right quadrant, LR) and late apoptotic cells as Annexin V- and PI-positive cells (upper right quadrant, UR). Apoptotic evaluation was calculated by the percentage of apoptotic cell number in total cell number (*P<0.05).

To determine whether apoptosis was contributing to the growth inhibition by sh-Rab14 vector, we performed Flow cytometry analysis of GC cells after transfection of GV-Rab14 or sh-Rab14 vector. In SGC-7901 cells, the rates of early (Annexin V-FITC+/PI-) and late apoptosis (Annexin V-FITC+/PI+) were lower than the control (Fig 4A). Meanwhile, there were also a significantly elevated rates of early or late apoptosis caused by Rab14 silencing in BGC-823 cells (Fig 4B). Together with previous experiments, these results suggested an essential contribution of Rab14 to the carcinogenesis of BGC-823 cells in GC progression.

### Rab14 promoted gastric cancer cell proliferation via AKT signaling pathway

**Fig 5:**
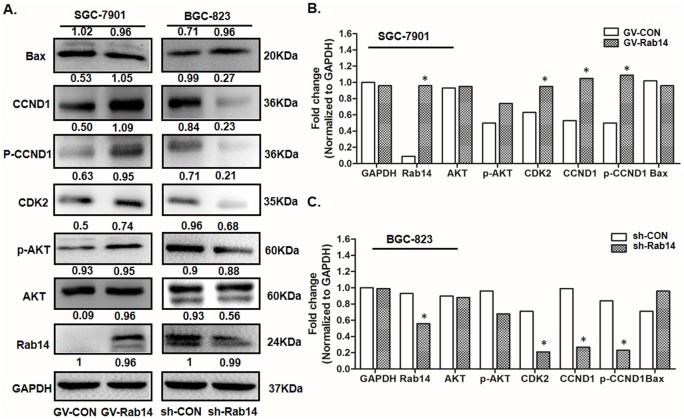
Rab14 affect gastric cancer cell progress through AKT signaling pathway. Expression analysis for AKT signaling pathway regulation proteins in SGC-7901 or BGC-823 cells by Western Blot. GAPDH was employed as a housekeeping control. Histogram represented the quantity analysis of the bands normalized to GAPDH (*P<0.05).

In order to further explore the possible molecular mechanisms of Rab14-mediated cell carcinogenesis progression, we detected the protein expression level of Rab14 and its downstream pathway regulators using Western blot. The PI3K/Akt pathway played important role in proliferation, migration and invasion of various cancer types, including gastric cancer [6]. Thus, we evaluated the effects of Rab14 on the Akt pathway by measuring the phosphorylation profile of Akt at Ser473. As is shown in Fig 5A, overexpression of Rab14 significantly activated the phosphorylation of Akt (Ser473) and knockdown of Rab14 dramatically inhibited the Akt (Ser473), whereas the protein expression of total AKT remained unchanged.

As our results have shown that Rab14 could induce G0/G1-S phase transition in GC cells, which prompted us to examine the underlying mechanisms of cell-cycle regulation. To conclude, overexpression of Rab14 could induce the expression of cdk2 and cyclinD1, and cause an activation at a phosphorylation of cyclin D1 at Thr286 in SGC-7901 cells (Fig 5B). Furthermore, silence of Rab14 in BGC-823 cells engendered a clear decrease of cdk2, cyclinD1 and phosphorylation of cyclin D1 (Thr286) (Fig 5C).

We also focused on the apoptosis of GC cells and examined whether Rab14 modified the downstream targets of PI3K related to apoptosis. As a result, overexpression of Rab14 dramatically inhibited the proapoptotic protein Bax and knockdown of Rab14 can promote apoptosis by accelerating Bax protein. These results demonstrate that Rab14 affects GC cell progression by modulating AKT pathways.

## Discussion

Gastric cancer remains one of the most common cancer types and is still a leading cause of cancer-related deaths. Its carcinogenesis is a multifactorial process related to cell proliferation [17], metastasis [18], and cell cycle [19]. At present, the molecular mechanisms underlying carcinogenesis and progression of GC have not been fully understood. Molecules involved in each step of the progress are potential prognostic and therapeutic markers [20]. Therefore, it’s of great importance to identify the role of unknown gene that could be useful in both patient prognosis and therapy [21]. In this study, we identified Rab14 as a candidate oncogene for GC growth and proliferation.

Rab14 is a member of the RAS oncogene family of small GTPase proteins, and RAB proteins have vital roles in vesicle trafficking, signal transduction and receptor recycling [22]. Much now understood about Rabs is function as regulatory GTPase through an activity regulation of downstream effectors like guanine exchange factors and GTPase activating proteins [23]. Previous reports demonstrated that Rab5-dependent endocytic transport of E- and N-cadherin was important for controlling cell-cell adhesions during vertebrate gastrulation and brain development, as well as receptor trafficking during the accompanying signaling events [24, 25]. Probably the best understanding of Rab11 family of GTPase-Rab4, Rab11, and Rab25 is that they are functioning in transporting different integrin complexes in migrating cells, in E-cadherin trafficking during adherens junction formation, and in cell polarization during asymmetric cell divisions [26–29]. Rab14 is the final member of Rab11 subfamily and was identified as oncogene negatively regulated by miR-451 or miR-338-3p at the posttranscriptional level via binding to 3‘-UTR in non-small cell lung carcinoma (NSCLC) cells [15, 30]. Although much has been learned about the role of Rab14 in the phagosomal systems and biosynthetic/recycling pathway, there were still little open-published studies regarding the expression and function of Rab14 in human cancer particular gastric cancer. Here, we investigated the expression of Rab14 in GC tumor tissues and cell lines. Compared to the matched non-tumor gastric tissues and normal gastric epithelial cell lines, the expression of Rab14 was up-regulated in both tumor tissues and cell lines which suggested that Rab14 might be a tumor oncogene in human GC.

In this study, we used SGC-7901 cells to overexpress Rab14, and used BGC-823 cells for Rab14 silencing to explore the function of Rab14 in GC. Both MTT and colony formation assays indicated that Rab14 could remarkably induce cell proliferation of GC cell lines. Moreover, Rab14 silencing with sh-Rab14 resulted in a marked cycle arrest from G1 to S transition and significantly elevated rates of early and late apoptosis in BGC-823 cells. The AKT signaling pathway is an important regulatory axis governing the cell proliferation or differentiation therefore is frequently deregulated in cancer [31]. Several studies have shown the importance of the AKT Kinase signaling pathway, which is constitutively active in gastric cancer and promotes cellular survival and tumorigenesis [32, 33]. As a key molecule effector of PI3K pathway, AKT play an important role in the regulation of cell cycle progression. Subbareddy et.al. described AKT could phosphorylates CDK2 at threonine 39 both in vitro and in vivo led a resulted in not only affect cell cycle transition but also changed subcellular localization promote apoptosis [34]. Thereafter, we investigated the possible molecular mechanisms of Rab14–induced cell carcinogenesis progression base on a potential perspective of AKT pathway. Rab14 Silencing in BGC-823 cells could reduce the expression of Rab14 protein and the phosphorylation of p-AKT at serine 473, whereas the protein expression of total AKT remained unchanged, which engendered a lower level of cyclinD1 and cdk2, and a clear decrease of cyclin D1 at a phosphorylation site in Thr286. In addition, AKT as the mediator plays an important role in apoptosis escape, it has been previously shown in Fuminori et.al.’s study that AKT could prevent Bax translocation from cytoplasma to mitochondria and revealed a novel mechanism which the AKT pathway promotes survival [35]. Similarly, in our study, we also provided evidence that suppression of p-AKT could promotes apoptosis by accelerating pro-apoptotic protein Bax. These results were exactly opposite to the effect of Rab14 overexpressing in SGC-7901 cells. Thus, we conclude that Rab14 can promote GC cells carcinogenesis through AKT signaling pathway, which is in agreement with previous research in human osteosarcoma U20S cells [36].

In summary, here we report the dysregulation expression of Rab14 in gastric cancer and found that Rab14 functions as an oncogene in gastric cancer cells by targeting through AKT pathway. This is a novel evidence to demonstrate the overexpression of Rab14 and may provide a new direction in prognosis or therapy of gastric cancer.

## Supporting Information

S1 Fig The uncropped and unaltered western blots used in this study. (TIF)
