# Supplementary figures and images for: Rab14 Act as Oncogene and Induce Proliferation of Gastric Cancer Cells via AKT Signaling Pathway
Source: PLoS One. 2017 Jan 20;12(1):e0170620. doi: 10.1371/journal.pone.0170620 (PMC5249107; doi:10.1371/journal.pone.0170620)

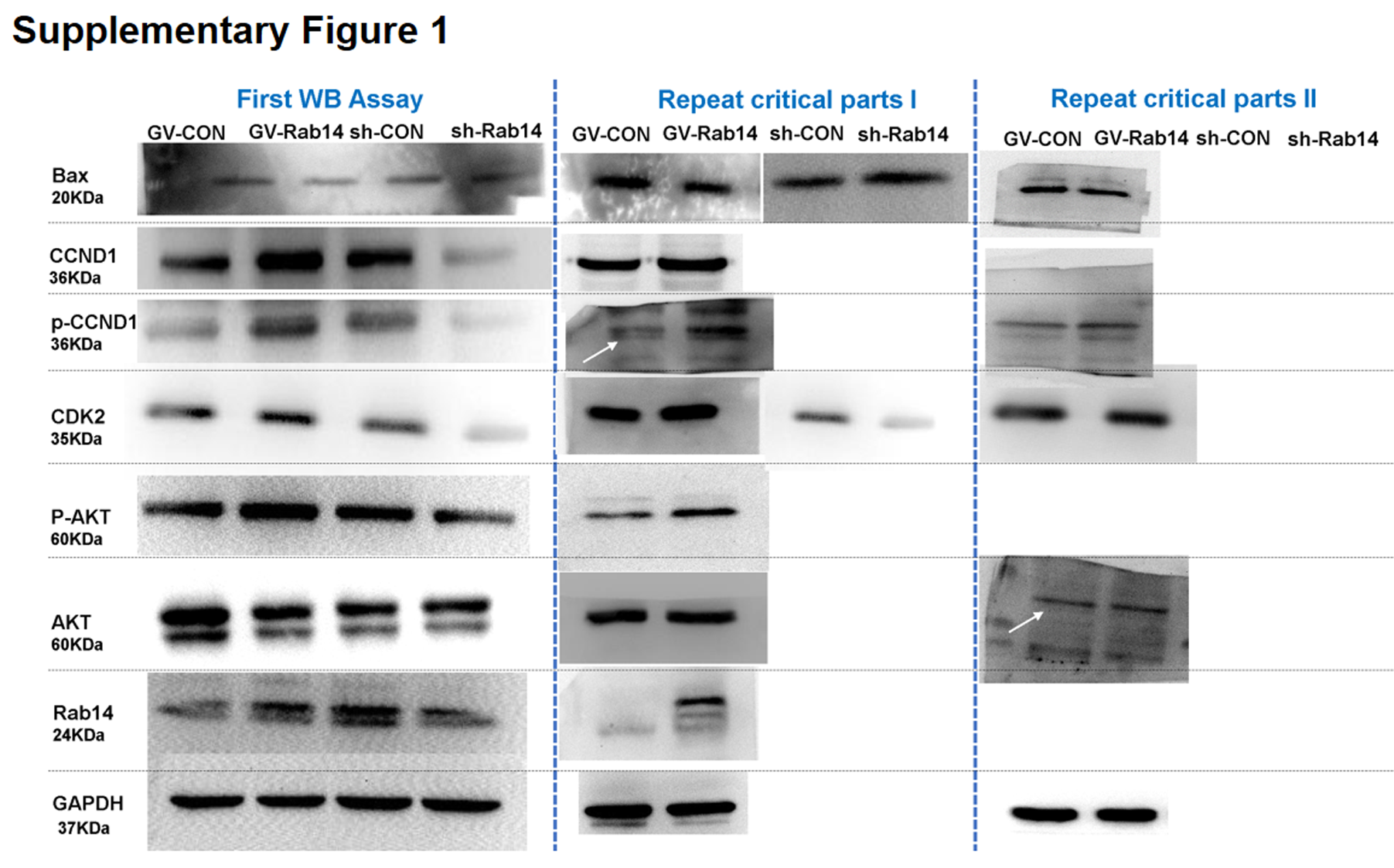

Supplement: S1 Fig — (TIF) [file pone.0170620.s001.tif]
